# Overexpression of mircoRNA-137 inhibits cervical cancer cell invasion, migration and epithelial–mesenchymal transition by suppressing the TGF-β/smad pathway via binding to GREM1

**DOI:** 10.1186/s12935-019-0852-8

**Published:** 2019-05-23

**Authors:** Hui Miao, Nuan Wang, Lin-Xin Shi, Zheng Wang, Wen-Bo Song

**Affiliations:** 1 Department of Radiotherapy, Xuzhou Cancer Hospital, Xuzhou, 221000 People’s Republic of China; 2 Department of Neurology, Xuzhou No. 1 People’s Hospital, Xuzhou, 221002 People’s Republic of China; 3 Clinical Medical College, Yangzhou University, Yangzhou, 225001 People’s Republic of China; 4 Department of Radiotherapy, Jiangdu People’s Hospital of Yangzhou, No. 9, Dongfanghong Road, Yangzhou, 225200 Jiangsu People’s Republic of China

**Keywords:** MicroRNA-137, Cervical cancer, GREM1, TGF-β/smad pathway, Epithelial–mesenchymal transition, Invasion, Migration

## Abstract

**Background:**

Accumulating evidence has highlighted the tumor suppressive roles of microRNA (miRNAs) in cervical cancer (CC). In the present study, we aim to delineate the functional relevance of microRNA-137 (miR-137) in influencing epithelial–mesenchymal transition (EMT), and other CC cell biological activities via the TGF-β/smad pathway by binding to GREM1.

**Methods:**

Microarray analysis was initially adopted to predict the differentially expressed genes and the miRNAs related to CC, followed by the measurement of the expression patterns of GREM1, EMT-related factors in the CC tissues and the adjacent tissues. Dual luciferase reporter gene assay was conducted to determine the relationship between miR-137 and GREM1. Gain-of- and loss-of-function experiments were conducted to characterize the effects of miR-137 and GREM1 on the colony formation, proliferation, apoptosis, migration, and invasion of CC cells in vitro, and the tumorigenicity of the CC cells in nude mice. The TGF-β/smad pathway was subsequently blocked with si-TGF-β to investigate its involvement.

**Results:**

Reduced miR-137 expression and increased GREM1 expression were predicted in CC, which was subsequently observed in the CC tissues and cells. Notably, GREM1 was a target gene of miR-137. The overexpressed miR-137 was found to inhibit EMT, cell proliferation, colony formation, invasion, migration and tumorigenesis in nude mice. In addition, miR-137 was noted to inhibit the activation of the TGF-β/smad pathway by binding to GREM1. The silencing of TGF-β1 was shown to reverse the effects induced by downregulated expression of miR-137.

**Conclusions:**

This study suggests that upregulated miR-137 suppresses the tumor progression in CC via blocking the TGF-β/smad pathway by binding to and negatively regulating GREM1.

**Electronic supplementary material:**

The online version of this article (10.1186/s12935-019-0852-8) contains supplementary material, which is available to authorized users.

## Background

Cervical cancer (CC) represents the fourth most frequently occurring malignancy among women across the globe and one of the leading causes of cancer-related death in women in developing countries [1]. Statistics data have reported approximately 266,000 deaths and 528,000 newly diagnosed cases of CC in Asian countries in 2010, among which about 6000 were found in China [2]. Many risk factors contributing to CC have been demonstrated, including polygamous spouse, early sexual activity, human papilloma virus history, smoking, as well as poor hygienism [3]. Owing to insufficient early detection, CC is usually diagnostically confirmed at a high-grade stage. Therefore, a growing body of studies have been identifying promising biomarkers to provide better understanding in underlying mechanism and improve the treatment outcome of CC [4].

CC-related expression profile (GSE63514) was obtained from GEO, which identified GREM1 as being highly expressed in CC. GREM1 has been well established to be an antagonist of bone morphogenetic protein, which is usually silenced by promoter hyper-methylation in human malignancies [5]. The functional relevance of GREM1 has been linked with the progression of multiple tumors, like colorectal cancer and basal cell carcinoma [6, 7]. Furthermore, transforming growth factor-β (TGF-β), acting as a multifunctional cytokine, which has been highlighted to influence malignant behaviors of tumor cells [8]. It is interesting to note that the TGF-β/smad pathway has also been verified as a critical mediator during the EMT process in CC [9]. The present study also demonstrated that GREM1 was actively involved in the regulaory process of TGF-β/smad pathway. However, its specific mechanism remains to be defined.

MicroRNAs (miRNAs) represent a family of small non-coding RNA molecules that could diminish the expression of corresponding target genes [10]. Notably, accumulating miRNAs have been identified to serve as both tumor suppressors and oncogenes to modulate CC progression due to its role in controlling metastasis as well as cell apoptosis [11]. The tumor suppressive role of miR-137 has been highlighted in a variety of cancers [12]. Accordingly, we conducted this study to validate the hypothesis that miR-137 may influence the expression of GREM1 and therefore controls the development of CC.

## Methods and materials

### Ethics statement

The study was conducted with the approval of the Institutional Review Board of Jiangdu People’s Hospital of Yangzhou (Number: 201402006). Written informed consent was obtained from each participant. The animal experiments are carried out in compliance with the *Guide for the Care and Use of Laboratory Animals* published by the National Institutes of Health.

### Microarray data and gene ontology (GO) enrichment analysis

CC-associated expression profiles were acquired from the Gene Expression Omnibus (GEO) database (http://lgmb.fmrp.usp.br/mirnapath/tools.php). A Limma package in the R language was employed to determine differentially expressed genes (DEGs) with screening criteria of |LogFoldChange| higher than 2 and *p* value less than 0.05. The heat-map package was adopted to plot the heat map. GO enrichment analysis was performed using the clusterProfiler, with *p *< 0.05 and gene counts > 2 as the screening criteria. The fold change of gene expression of top 50 genes is displayed in Additional file 1, and the gene expression value of top 50 genes in Additional file 2.

### Regulatory miRNA prediction

MiRDB database (http://mirdb.org/miRDB/index.htmL), mirDIP database (http://ophid.utoronto.ca/mirDIP/index.jsp#), DIANA database (http://diana.imis.athena-innovation.gr/DianaTools/index.php?r=microT_CDS/index) and miRNApath database (http://lgmb.fmrp.usp.br/mirnapath/tools.php) were adopted to identify the regulatory miRNAs for GREM1. Afterwards, we intersected the results from the four databases to predict the regulatory miRNAs of GREM1 by the Venn plotting website (http://bioinformatics.psb.ugent.be/webtools/Venn/), after which the miRNA with the highest credibility was obtained.

### Patients

From March 2014 to March 2016, 109 CC patients (24–71 years old; average age = 44.34 years) from the Department of Gynecology and Obstetrics of Jiangdu People’s Hospital of Yangzhou were recruited for this study. None of the patients had undergone chemotherapy, radiotherapy or biotherapy prior to the surgery. The patients were enrolled if the following criteria were met: (1) CC diagnosis confirmed following histopathological examinations, based on the diagnostic standard of the sixth edition of *Obstetrics and Gynecology*; (2) CC staging determined based on classification criteria recommended by International Federation of Gynecology and Obstetrics (FIGO) [13]. Women who requested or required a radical hysterectomy were also included, the diagnosis of whom was verified by pathological examination after surgery. Patients were excluded if they: (1) had incomplete clinical data; (2) suffered from congenial acute genital tract inflammation, genetic diseases, or any other serious internal medical diseases, or pregnant women. Adjacent tissues 2 cm from the CC tissues, which were pathologically confirmed as normal tissues, were collected to serve as controls.

### Immunohistochemistry

The immunohistochemistry was conducted based on the protocols on the SP Kit (wi83516, Beijing Ruoshuihe Technology Co., Ltd., Beijing, China) [14]. Both CC and the adjacent tissues were prepared into paraffin-embedded sections. Phosphate buffered saline (PBS) and a known positive section were separately served as a negative control (NC) and a positive control. Following dewaxing, the sections were subjected to the treatment of citrate buffer for antigen repair. After treatment of endogenous peroxidase blocking, the sections were cultured with primary antibodies, rabbit antibodies to GREM1 (1:100; ab22138), E-cadherin (1:30, ab15148), N-cadherin (1:200, ab18203) and Vimentin (1:100, ab16700). All the antibodies were provided by Abcam Inc. (Cambridge, MA, USA). Subsequently, biotin-labeled secondary antibody was employed to incubate the sections. The proteins were visualized by diaminobenzidine (GMS12048.1, Shanghai Genmed Gene Pharmaceutical Technology Co., Ltd., Shanghai, China). The cells were counted (over 200 cells) in 4 randomly selected view fields using an optical microscope and protein expression rate was determined as positive cells/total cells × 100%.

### Dual-luciferase reporter gene assay

The putative binding sites between miR-137 and GREM1 were identified with the bioinformatics website (microRNA.org). The pmirGLO plasmids (Promega Corporation, Madison, WI, USA), GREM1 3′untranslated region (UTR) and 3′UTRmu fragments were inserted through *Xba*I and *Sac*I. The wild-type or mutant reporter constructs were co-transfected into the plated *Escherichia coli* DH5α cells (Takara, Dalian, Liaoning, China). When the cell reached 90–95% confluence, GREM1 3′UTR-pmir-GLO, 3′UTRmu-pmirGLO, or miR-137 mimic (GenePharma Ltd., Shanghai, China) or miR-137 mimic NC were transfected using Lipofectamine 2000 transfection (Invitrogen Inc., Carlsbad, CA, USA). The light intensity was determined based on the protocols of Dual-Luciferase^®^ Reporter Assay System (Promega). In order to prevent the off-target effects, GREM1 3′UTR-pmir-GLO was co-transfected with specific miR-137 inhibitor and miR-137 mimic. After 24, the cells transfected with an inhibitor were regarded as the NC group, while those without any transfection as the blank control. The assay was independently repeated 3 times.

### Cell lines and co-culture conditions

Human normal immortalized epithelial cell line HaCaT and CC cell lines C33A, HeLa, Caski and Siha (CL-0210, Wuhan Procell Life Technology Co., Ltd., Wuhan, Hubei, China) were cultured in RPMI-1640 supplemented with 10% fetal bovine serum (FBS) at 37 °C with 5% CO_2_. The cells were then assigned into six groups to investigate the effect of miR-137 binding to GREM1 on behaviors of CC cells, namely: blank (without transfection); NC (transfected with NC), miR-137 mimic (transfected with miR-137 mimic), miR-137 inhibitor (transfected with miR-137 inhibitor), siRNA-GREM1 (transfected with siRNA-GREM1 sequence) and miR-137 inhibitor + si-GREM1 (co-transfected with miR-137 inhibitor + si-GREM1). To investigate the effect of the TGF-β/smad pathway mediated by miR-137 on behaviors of CC cells, the cells were also tansducted with miR-137 inhibitor + si-NC or miR-137 inhibitor + si-TGF-β. 24 h prior to transfection, the cells were plated into a 6-well plate, after which the transfection was carried out based on the protocols of lipofectamine 2000 (11668-019, Invitrogen) when the cells reached 30–50% confluence. After incubation for 6–8 h, the medium was renewed with complete medium. After further incubation for 24–48 h, the cells were harvested and reserved.

### RNA extraction and RT-qPCR

The Trizol (Takara) method was adopted for obtaining total RNA from the tissues. The sample RNA was reversely transcribed into cDNA using a reverse transcription kit (Fermentas K1621, Hangzhou ZhuNuo Biotechnology, Hangzhou, China). The primers used for amplification were artificially synthesized by TaKaRa (Table 1). Fluorescent quantitative PCR was performed based on the protocols of the SYBR^®^ Premix Ex Taq™ II Kit (RR820A, XingZhi Biotch. Guangzhou, China) using the ABI PRISM^®^ 7300 (Shanghai Kunke Instrument and Equipment Co., Ltd., Shanghai, China). U6 was regarded as the housekeeping gene for miR-137, and GAPDH as internal control for the remaining genes. The relative mRNA expression was quantified based on the 2^−ΔΔCt^ method. The aforementioned methods were also applicable to cell assays.

**Table 1:** Primer sequences of miR-137, U6, GREM1, TGF-β1, Smad2, Smad3 and Smad4 used in RT-qPCR

Gene	Primer sequences (5′–3′)
miR-137	F: TTATTGCTTAAGAATACGCGTAG
	R: TGGTGTCGTGGAGTCG
U6	F: GCTTCGGCACATATACTAAAAT
	R: CGCTTCACGAATTTGCGTGTCAT
GREM1	F: TAACACTGCCACAAGAATGCAA
	R: GCAAGACTGTGGTACAAGCTCCTAA
TGF-β1	F: GCTCCACGGAGAAGAACAGGCTG
	R: CTGCTCCACCTTGGGCTTGC
Smad2	F: CGAAATGCCAGCGTAFAAAT
	R: CTGCCTTCGGTATTCTGCTC
Smad3	F: GCCCGTTACCTACTCGGAGC
	R: TGTTGACATTGGAGAGCAGC
Smad4	F: ATGCCTGTCTAGGCATTGTG
	R: CTGAAGCCTCCCATCCAAT
GAPDH	F: TGTGGGCATCAATGGATTTGG
	R: ACACCATGTATTCCGGGTCAAT

*RT-qPCR* reverse transcription quantitative polymerase chain reaction, *miR-137* microRNA-137, *GREM1* gremlin-1, *F* forward, *R* reverse, *TGF-β1* transforming growth factor β1, *GAPDH* glyceraldehyde-3-phosphate dehydrogenase

### Western blot analysis

The extraction of total protein was carried out with RIPA lysis buffer. In the cellular protein extraction, the cells were lysed with 400 μL of lysis buffer for 30 min on ice. Subsequently, the cells were centrifuged to obtain the supernatant. The proteins were separated by electrophoresis and then transferred onto a polyvinylidene fluoride (PVDF) membrane, which was then blocked by 5% bovine serum at room temperature for 1 h. The membrane was then incubated at 4 °C overnight with the primary antibodies, diluted monoclonal rabbit antibodies to GREM1 (#4383, 1:1000, Cell Signaling Technology, Beverly, MA, USA), Smad4 (ab40759, 1:5000), the mouse antibodies to TGF-β1 (ab64715, 1:1000), Smad2 (ab119907, 1:1000), Smad3 (ab75512, 1:5000), E-cadherin (ab1416, 1:50), N-cadherin (ab98952, 1:1000), Vimentin (ab8978, 1:1000) and β-actin (ab8226, 1:1000). The membrane was subsequently incubated with corresponding secondary antibody, goat anti-rabbit antibody (ab6721, 1:5000) or goat anti-mouse antibody (ab6789, 1:5000) for 1 h at room temperature. Next, proteins were visualized with an enhanced chemiluminescence kit and observed by a Gel Imaging System for gray value analysis. The experimental procedures were also applicable for the tissue detection.

### Cell viability assay

Transfected CC cells in the exponential phase were prepared into cell suspension. The cells (1 × 10^4^ cells for each well) were then plated in a 96-well plate with 180 μL of cell suspension per well, followed by culture at 37 °C and 5% CO_2_ for 24–72 h. The plate was incubated for 4 h with 5% MTT solution (20 μL/well) without light exposure at 37 °C and 5% CO_2_. After this, the plate was treated with dimethyl sulfoxide (DMSO, 100 μL/well) in the dark for dissolving the crystal. Subsequently, the optical density (OD) was tested using a microplate reader (SAF-680T, Multiskan GO, Thermo Fisher Scientific, USA) at a wavelength of 570 nm. Growth curves of cells were plotted with the time as the X-axis and OD as the Y-axis. The assay was independently conducted three times.

### Colony formation assay

Following cell transfection for 72 h, the cells at the exponential phase were seeded in 6 well plates (200 cells/well) respectively for a 2-week incubation. Each group was repeated in six wells. The culture was terminated when white clone spots were observed with the naked eye. The cells were fixed in methanol and stained with Giemsa. The colonies formed were observed under an optical microscope with a mass of more than 50 cells as one colony. The plating efficiency was calculated using the following formula: colony formation rate = clones counted/cells plated × 100%. The assay was repeated 3 times independently.

### Flow cytometric analysis

After cells plated in 6-well culture plates adhered to the well, the cells were synchronized for 12 h. Digested cells were harvested after centrifugation, resuspended with pre-cooled 75% ethanol and fixed overnight at − 20 °C. The cells were incubated with a 50 μL aliquot of propidium iodide (PI; 0.5 mg/mL), and water-bathed at 37 °C for 30 min. The cell cycle distribution was assessed using a flow cytometer (FACSCalibur; Becton–Dickinson, Franklin Lakes, NJ, USA). The assay was independently repeated 3 times.

The cell apoptosis was evaluated based on the protocols of the Annexin-V-FITC Kit (Bender MedSystems, Vienna, Austria). The cultured cells were prepared into a single cell suspension with 0.05% trypsin. The cells were pipetted to obtain cell suspension. Subsequently, the cells were incubated with Annexin-V-FITC, and then further incubated with PI for 5 min under dark conditions. A flow cytometer was adopted to measure cell apoptosis rate.

### Matrigel invasion assay

The Transwell apical chamber was coated with Matrigel diluted with DMEM to 50 μg/mL. The cells at exponential phase were then collected and photographed to analyze cell invasion at the 0th h. After an incubation process for 24 h, the cells were then photographed again. Fold changes of cell invasion were calculated. Matrigel diluted by serum-free medium was employed in the Transwell apical chamber to cover the polycarbonate membrane, which was left to react at room temperature for 1 h. Next, DMEM containing 20% FBS was applied to the Transwell basolateral chamber. The transfected CC cells for 24 h were prepared into single cell suspension by the serum-free DMEM (5 × 10^5^ cells/mL), which was added to each well and cultured for 24 h. The liquid in the apical chamber was discarded and the cells that failed to penetrate through the membrane were wiped off. After hematoxylin–eosin–methylene staining, the number of cells penetrating the membrane was counted from 3 randomly selected fields by a light microscope. The assay was independently repeated 3 times.

### Scratch test

The cells were trypsinized at 37 °C until the cell layer adhering to the well was noted to be moving in a sand like manner. The cells were seeded in a 6-well plate and incubated with 10% FBS overnight. When cell confluence reached 90–100%, vertical linear scratches were made at the bottom of the plate using a micropipette tip with 4–5 scratches in each well. The width of each scratch was made to be identical. Afterwards, the cells were incubated with serum-free medium again. The migration distance was observed and photographed under an inverted microscope at the 0th h and 24th h after scratching. IPP7.0 software (Media Cybernetics Inc., Singapore Post Centre, Singapore) was adopted to calculate the cell-free area at each time point, which was compared with that at the 0th h to calculate the migration rate. Six replicates were set up for each group. The assay was independently repeated 3 times.

### Tumor formation in nude mice

Sixty female BALB/C athymic nude mice (6-week-old, Shanghai SLAC Laboratory Animal Co., Ltd., Shanghai, China) were randomly divided into 6 groups: blank, NC, miR-137 mimic, miR-137 inhibitor, siRNA-GREM1 as well as miR-137 inhibitor + si-GREM1. The nude mice were fed under a thermostatic and pathogen-free environment, which were observed approximately once a week prior to treatment. The transfected cells, after digestion, were centrifuged at 1000 rpm for 5 min. The cells were harvested and mixed in diluted Matrigel. The cell suspension (5 × 10^7^ cells per mice) was subcutaneously injected into the left axilla of the nude mice. Afterwards, the weight of nude mice was recorded every day, and the volume was recorded every 5 days using Vernier calipers, with growth curves plotted. After 40 days, the nude mice were euthanized by CO_2_ inhalation and photographed. The tumor weight and size were measured, and the tumor volume was all determined.

### Statistical analysis

Statistical analysis was carried out using the SPSS 21.0 software (IBM Corp., Armonk, N.Y., USA). The enumeration data were presented by rate or percentage, and processed by χ^2^ test, and the measurement data by the mean ± standard deviation. Differences between CC tissues and adjacent tissues were compared by paired *t* test, while other pairwise comparisons were performed using independent sample *t* test. Differences among multiple groups were compared with one-way analysis of variance. Spearman rank correlation analysis was adopted for correlation analysis. Statistical significance was defined at a level of 5% (*p* < 0.05).

## Results

### miR-137 was predicted to participate in the progression of CC via regulating GREM1

**Fig. 1:**
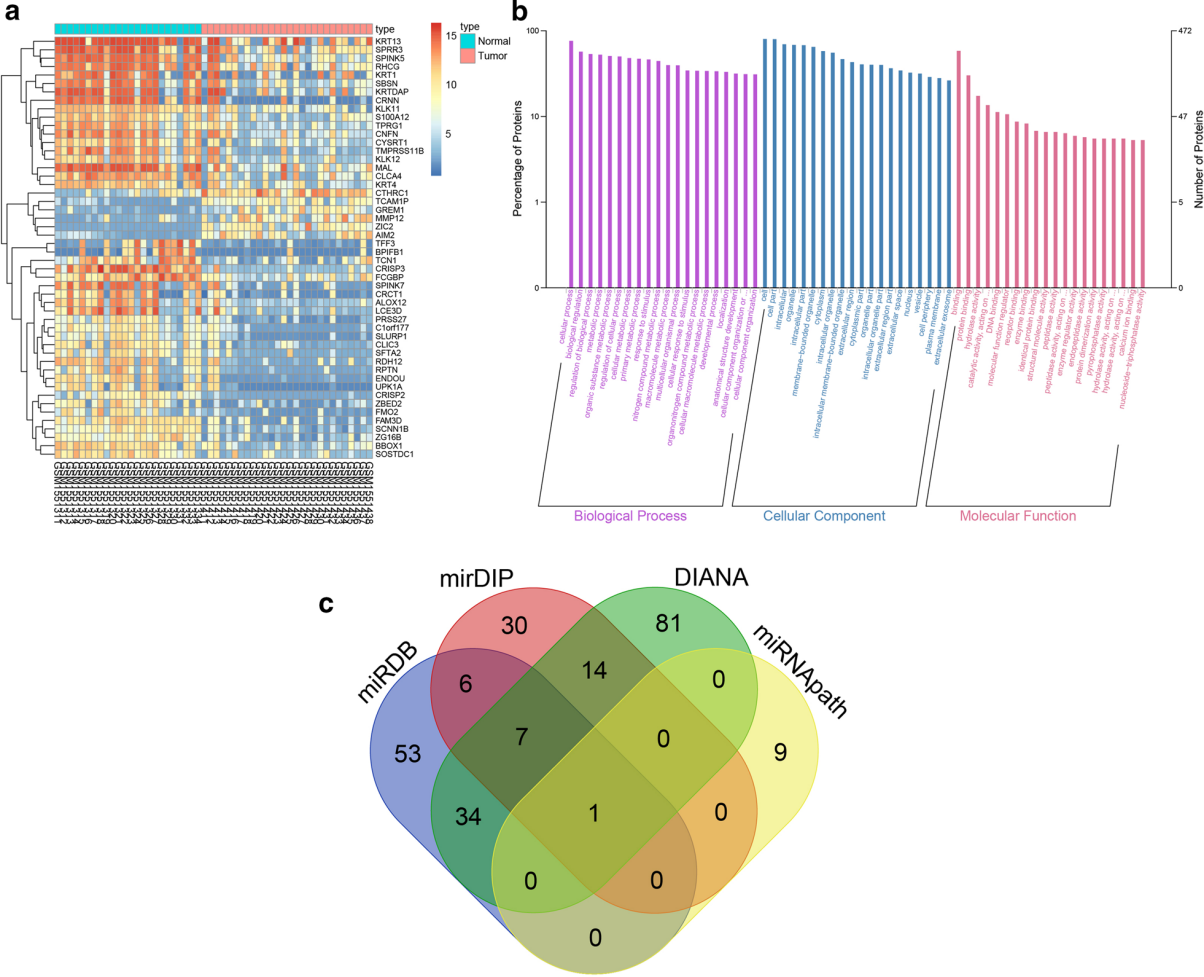
miR-137 is predicted to regulate the progression of CC via TGF-β1/smad pathway by binding to GREM1. **a** The heat map of the top 50 genes with the highest differential expression where the X-axis represents the sample number, the Y-axis represents genes. The left dendrogram indicates gene expression cluster in which each small square in the graph represents the expression of a gene in one sample, and the histogram of the upper right is the color gradation in which blue stands for low expression and red stands for high expression; **b** GO analysis of DEGs of GSE63514; the X-axis indicates the name of GO items, the Y-axis reflects the percentage of genes, the grape area represents biological process clustering, the blue represents cellular component clustering, the red signifies molecular function clustering; **c** the prediction result of miRNAs regulating GREM1 gene; the four different color ellipses represent the number of miRNAs regulating GREM1 in miRDB, mirDIP, DIANA, and miRNApath databases, and the overlapping section represents the intersection of each other, the central part represents the common intersection of 4 databases. *miR-137* microRNA-137, *CC* cervical cancer, *GREM1* gremlin-1, *GO* gene ontology, *DEGs* differentially expressed genes

The miRNAs associated with CC were retrieved by analyzing GSE63514 (https://www.ncbi.nlm.nih.gov/geo/query/acc.cgi?acc=GSE63514) obtained from the GEO database. Afterwards, we conducted differential expression analysis of genes between 24 normal controls and 28 CC tissues. Next, 494 DEGs were obtained, in which 177 DEGs were identified with high expression in CC tissues, and the remaining 317 DEGs with poor expression in CC tissues, relative to normal controls. As shown in Fig. 1a, the heat map of the top 50 DEGs was plotted with the most significant difference in the 494 DEGs (Addtional files 1, 2). GO enrichment for those DEGs revealed that the DEGs mainly participates in the biological regulation in the biological process, cell part in, cellular component, and protein biding in molecular function, respectively, which were speculated to be correlated to CC (Fig. 1b). A growing body of studies have reported the key functional relevance of GREM1 in neoplastic diseases, which was among the genes exhibiting the most significant differential expression [15–17]. However, there was very little literature demonstrating the mechanism of GREM1 in CC. Moreover, some studies have revealed that GREM1 is most likely to participate in the regulation of the TGF-β pathway, which has been verified as an essential pathway involved in the progression of CC [18–20]. The differential expression analysis of GSE63514 identified a notable upregulation of GREM1 level in CC tissues. Thus, GREM1 was selected for subsequent experiments. In an attempt to acquire further knowledge in the upstream regulatory mechanism of GREM1, such databases as miRDB, mirDIP, DIANA, miRNApath were adopted to predict regulatory miRNAs, followed by intersecting the prediction results. The intersection determined the only regulatory miRNA miR-137 in the four databases (Fig. 1c).

### GREM1 is highly expressed and EMT is increased in CC tissues

**Fig. 2:**
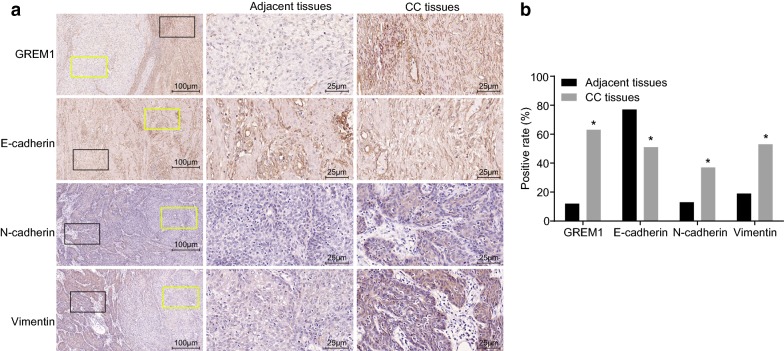
EMT and GREM1 expression are induced in CC tissues. **a** Immunohistochemical staining of GREM1, E-cadherin, N-cadherin and Vimentin in the adjacent and CC tissues (scale bar = 100 um; black square is CC tissue, and yellow square is adjacent tissue); **b** positive rate of GREM1, E-cadherin, N-cadherin and Vimentin in the adjacent and CC tissues; the protein expression = positive cells/total cells × 100%, the experimental data were expressed as percentage and analyzed by χ^2^ test; n = 109; *p < 0.05 vs. the adjacent tissues. *GREM1* gremlin-1, *EMT* epithelial–mesenchymal transition, *CC* cervical cancer

The expression of GREM1, E-cadherin, N-cadherin and Vimentin in CC tissues and adjacent tissues was examined using immunohistochemistry (Fig. 2a, b), and the positive expression was presented as brown particles. GREM1 was predominantly expressed in the cell membrane and cytoplasm. GREM1 was poorly or barely expressed in the adjacent tissues (11.9%), and the expression rate was increased in CC tissues (63.3%) (*p* < 0.05). E-cadherin was predominantly expressed in the cell membrane and cytoplasm, with a expression rate in the adjacent tissues as 77.1% and a lower rate in CC tissues as 51.4% (*p* < 0.05). N-cadherin and Vimentin, mainly expressed in the cytoplasm, were negatively expressed in adjacent tissues with expression rates of 12.8% and 19.3%, respectively. Besides, their expression rates (36.7% and 53.2%) in CC tissues were obviously increased (*p* < 0.05). Furthermore, the correlation between GREM1 and EMT-related proteins (E-cadherin, N-cadherin and Vimentin) was analyzed by Spearman rank correlation analysis. The expression of GREM1 was inversely correlated with E-cadherin expression (r = − 0.512, *p* < 0.001). Positive correlation was identified between the expression of GREM1 and the expression of N-cadherin and Vimentin (r = 0.580, *p* < 0.001; r = 0.545, *p* < 0.001).

### Lower miR-137 expression, higher GREM1 expression and activated TGF-β/smad pathway in CC tissues

**Fig. 3:**
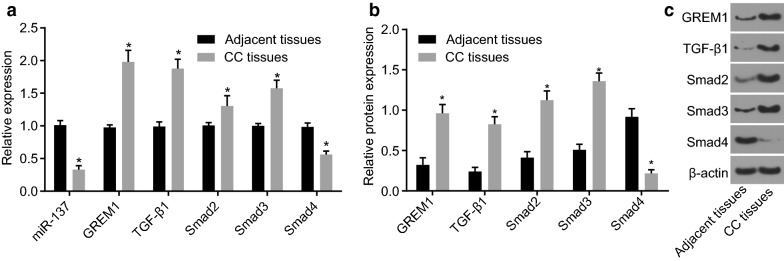
CC tissues presented with lower miR-137 expression, higher GREM1 expression and activated TGF-β1/smad pathway. **a** The expression of miR-137 and mRNA expression of GREM1, TGF-β1, Smad2, Smad3 and Smad4 in CC tissues detected by RT-qPCR normalized to adjacent tissues; **b**, **c** the protein expression of GREM1, TGF-β1, Smad2, Smad3 and Smad4 examined by Western blot assay normalized to β-actin; data were presented as mean ± standard deviation and analyzed by paired t-test; n = 109; *p < 0.05 vs. adjacent tissues. *miR-137* microRNA-137, *GREM1* gremlin-1, *RT-qPCR* reverse transcription quantitative polymerase chain reaction

The expression of the potential tumor suppressor miR-137, GREM1 and the TGF-β/smad pathway-related genes in tissues were determined. Relative to the adjacent tissues, the expression of miR-137 was lower in the CC tissues (*p* < 0.05), while the expression of GREM1, TGF-β1, Smad2, and Smad3 was higher, accompanied by lower expression of Smad4 (*p* < 0.05; Fig. 3a–c). Accordingly, in the CC tissues, there was an abnormal activation of the TGF-β/smad pathway, along with activated TGF-β1, which induced the activation of Smad2 and Smad3. However, the low expression of Smad4 was able to reduce the inhibitory effect of TGF-β1, which promoted the growth of CC tumor.

### MiR-137 and GREM1 may be involved in the clinicopathological features of CC

The expression of miR-137 and GREM1 and their relationship to clinicopathological features were further investigated in CC tissues (Table 2). No direct correlation was revealed between the expression of miR-137 and that of GREM1 in CC tissues with age of patients suffering with CC (*p *> 0.05). The miR-137 expression in CC patients at FIGO stage < II was higher than that of the CC patients at stage ≥ II (*p *< 0.05), while the mRNA expression of GREM1 in the CC patients at FIGO stage < II was lower than that of CC patients at stage ≥ II (*p *< 0.05). The expression of miR-137 in patients without lymph node metastasis was elevated versus patients with lymph node metastasis (*p *< 0.05), while its correlation with the expression of GREM1 was inverse. There was a higher expression in miR-137 in patients without vascular infiltration in comparison to that of patients with vascular infiltration (*p* < 0.05). However, its correlation with the mRNA expression of GREM1 was opposite to the aforementioned findings. The expression of miR-137 in patients with higher differentiation degree was higher than that of patients with lower differentiation degree (*p* < 0.05); besides, an inverse trend was observed in its correlation with the expression of GREM1. The expression of miR-137 in cases with a tumor size ≤ 4 cm was higher than that of cases with tumor size > 4 cm. The expression of GREM1 was determined to have exerted an opposite effect. These findings revealed miR-137 and GREM1 were involved in the development of CC.

**Table 2:** Correlation of miR-137 and GREM1 with clinicopathological features of cervical cancer

Clinicopathological features	n	miR-137 expression	*p*	GREM1 mRNA expression	*p*
Age (years)					
≤ 40	57	0.332 ± 0.066	0.798	1.984 ± 0.195	0.860
> 40	52	0.329 ± 0.055		1.978 ± 0.156	
Stage					
< II stage	57	0.347 ± 0.064	0.002	1.938 ± 0.174	0.008
≥ II stage	52	0.312 ± 0.052		2.028 ± 0.170	
Lymph node metastasis					
No	72	0.341 ± 0.065	0.011	1.951 ± 0.165	0.012
Yes	37	0.310 ± 0.046		2.040 ± 0.187	
Vascular infiltration					
No	65	0.346 ± 0.070	0.001	1.950 ± 0.199	0.025
Yes	44	0.307 ± 0.032		2.027 ± 0.128	
Differentiation degree					
Poorly differentiated	31	0.302 ± 0.062	< 0.001	2.084 ± 0.191	< 0.001
Moderately differentiated	43	0.326 ± 0.061		1.994 ± 0.174	
Well-differentiated	35	0.361 ± 0.043		1.874 ± 0.090	
Tumor size (cm)					
≤ 4	60	0.349 ± 0.063	< 0.001	1.928 ± 0.163	< 0.001
> 4	49	0.308 ± 0.049		2.046 ± 0.174	

Statistical data were presented as mean ± standard deviation; data between two groups were analyzed by paired *t*-test and comparisons among multiple groups were analyzed by one-way analysis of variance

*miR-137* microRNA-137, *GREM1* gremlin-1

### GREM1 is validated as a target gene of miR-137

**Fig. 4:**
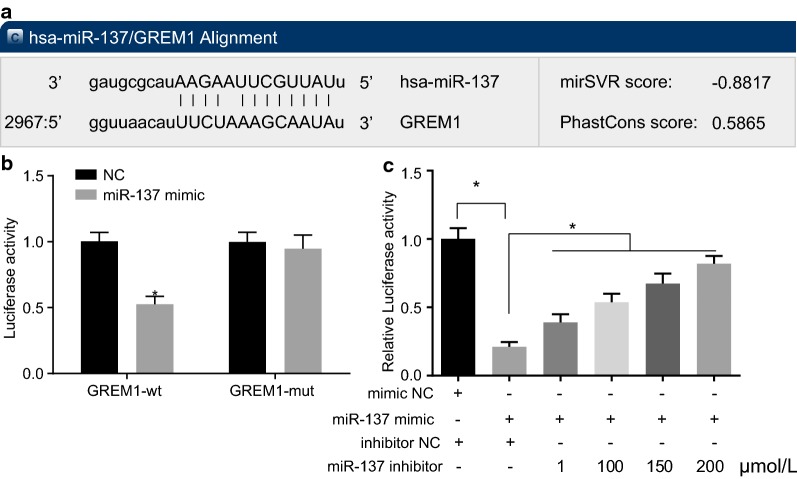
GREM1 is a target gene of miR-137. **a** Prediction of binding sites between miR-137 and GREM1 identified by bioinformatics website; **b**, **c** dual-luciferase reporter assay for confirmation of the targeting relationship between miR-137 and GREM1; *p < 0.05 vs. the NC group; statistical data were presented as mean ± standard deviation; data between two groups were analyzed by paired *t*-test; one-way analysis of variance was used for multi-group comparisons; the experiment was repeated 3 times independently. *GREM1* gremlin-1, *miR-137* microRNA-137, *NC* negative control

The biological prediction website (microRNA.org) presented evidence suggesting that miR-137 targets GREM1 (Fig. 4a). Based on the analysis of dual-luciferase reporter gene assay, luciferase activity was decreased in the cells co-transfected with GREM1-wt and miR-137 mimic in the miR-137-Wt group, relative to the NC group (*p *< 0.05). No notable difference was found in the luciferase activity in cells co-transfected with GREM1-Mut and miR-137 mimic (*p* > 0.05; Fig. 4b). Following co-transfection of miR-137 inhibitor, miR-137 mimic and GREM1 3′UTR-pmir-GLO, luciferase activity increased gradually with the elevation of miR-137 inhibitor concentration (Fig. 4c). The above-mentioned results confirmed expression of GREM1 in cells was affected by miR-137 expression. There is an obvious negative correlation between GREM1 and miR-137. Based on the above results, GREM1 is a target gene of miR-137. MiR-137 could target GREM1 and inversely regulate its expression.

### The expression of miR-137 and GREM1 in CC cells

**Fig. 5:**
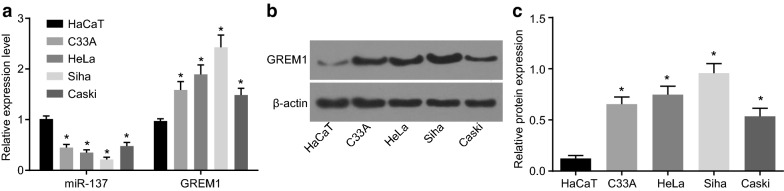
The Siha cell line exhibits the lowest expression of miR-137 and the highest expression of GREM1. **a** The miR-137 expression and mRNA expression of GREM1 in Caski, HeLa, C-33A and Siha cell lines normalized to HaCaT cells; **b** the gray value of GREM1 protein band in HaCaT, Caski, HeLa, C-33A and Siha cell lines normalized to β-actin; **c** the protein expression of GREM1 in HaCaT, Caski, HeLa, C-33A and Siha cell lines; *p < 0.05 vs. the normal epithelial cell line HaCaT; statistical data were presented as mean ± standard deviation; one-way analysis of variance was used for multi-group comparisons; the experiment was repeated 3 times independently. *miR-137* microRNA-137, *GREM1* gremlin-1

The expression of miR-137 and the mRNA expression of GREM1 were measured with the use of RT-qPCR (Fig. 5a) in the CC cell lines and the normal epithelial cells, where the protein expression of GREM1 was determined with Western blot analysis (Fig. 5b, c). The expression of miR-137 in CC cell line C33A (HPV negative cervical cancer cells, carcinoma), HeLa (HPV-18 infected cervical cell lines, adenocarcinoma), Caski (HPV-16 infected cervical cancer cell lines, epidermoid carcinoma), and Siha (HPV-16 infected cervical cancer cell lines, grade II squamous cell carcinoma) was notably lower than that in the normal epithelial cell line HaCaT (*p* < 0.05). The relative expression of miR-137 was 0.480 ± 0.072 in Caski, 0.352 ± 0.051 in HeLa, 0.448 ± 0.063 in C-33A and 0.213 ± 0.047 in Siha with its expression set as 1 in HaCaT cell line. Furthermore, the expression of miR-137 was the lowest in the Siha cells and the highest in the Caski cells. The expression of GREM1 in CC cell lines Caski, HeLa, C-33A, and Siha was higher than that in the HaCaT cells (*p* < 0.05). GREM1 expression was the highest in the Siha cells and lowest in the Caski cells. Accordingly, we selected the cell line Siha for subsequent experiments.

### Overexpression of miR-137 represses the activation of TGF-β/smad pathway and EMT by binding to GREM1

**Fig. 6:**
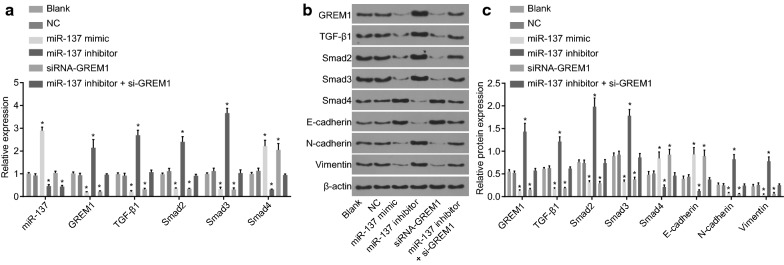
Overexpression of miR-137 inhibits EMT and the activation of TGF-β/smad pathway by binding to GREM1 in CC. **a** The miR-137 expression and the mRNA expression of GREM1, TGF-β1, Smad2, Smad3 and Smad4 in CC cells treated with miR-137 mimic, inhibitor and/or siRNA-GREM1 examined by RT-qPCR normalized to cells without transfection; **b**, **c** the protein expression and protein bands of GREM1, TGF-β1, Smad2, Smad3, Smad4, E-cadherin N-cadherin and Vimentin in cells treated with miR-137 mimic, miR-137 inhibitor and/or siRNA-GREM1, normalized to β-actin; *p < 0.05 vs. the blank and NC group; statistical data were presented as mean ± standard deviation; one-way analysis of variance was used for multi-group comparisons; the experiment was repeated 3 times independently. *EMT* epithelial–mesenchymal transition, *CC* cervical cancer, *GREM1* gremlin-1, *miR-137* microRNA-137, *TGF-β1* transforming growth factor β1, *NC* negative control

We have already found that miR-137 was lowly expressed in CC cell, but the specific mechanism remains unclear. RT-qPCR and western blot analysis (Fig. 6a–c) revealed that no significant difference was identified in the expression of miR-137 and the mRNA and protein level of GREM1, TGF-β1, Smad2 and Smad3 between the blank and NC groups (all *p* > 0.05). Versus the blank and NC groups, the expression of miR-137 showed no significant change in the siRNA-GREM1 group (*p* > 0.05), while it was increased in the miR-137 mimic group (*p *< 0.05) and decreased in the miR-137 inhibitor group and the miR-137 inhibitor + si-GREM1 group (*p* < 0.05). In comparison with the blank and NC groups, the mRNA and protein level of GREM1, TGF-β1, Smad2, Smad3, N-cadherin, and Vimentin presented with no significant difference in the miR-137 inhibitor + si-GREM1 group (*p* > 0.05), while the miR-137 inhibitor group presented with a notable elevation (*p* < 0.05), and the miR-137 mimic and siRNA-GREM1 groups were found with a significant reduction (*p* < 0.05). The mRNA and protein level of Smad4 and E-cadherin in the miR-137 inhibitor + si-GREM1 group were not significantly different (*p* > 0.05), but enhanced in the miR-137 mimic group and the siRNA-GREM1 group (*p* < 0.05), and diminished in the miR-137 inhibitor group (*p* < 0.05), relative to the blank and NC groups. These results suggested that the abnormal activation of the TGF-β/smad pathway in CC cells resulted in the overexpression of TGF-β1, Smad2 and Smad3 and low expression of Smad4, which induced EMT in CC cells with down-regulated expression of E-cadherin, and up-regulated N-cadherin and Vimentin expression.

### Overexpression of miR-137 reduces CC cell proliferation by inhibiting GREM1

**Fig. 7:**
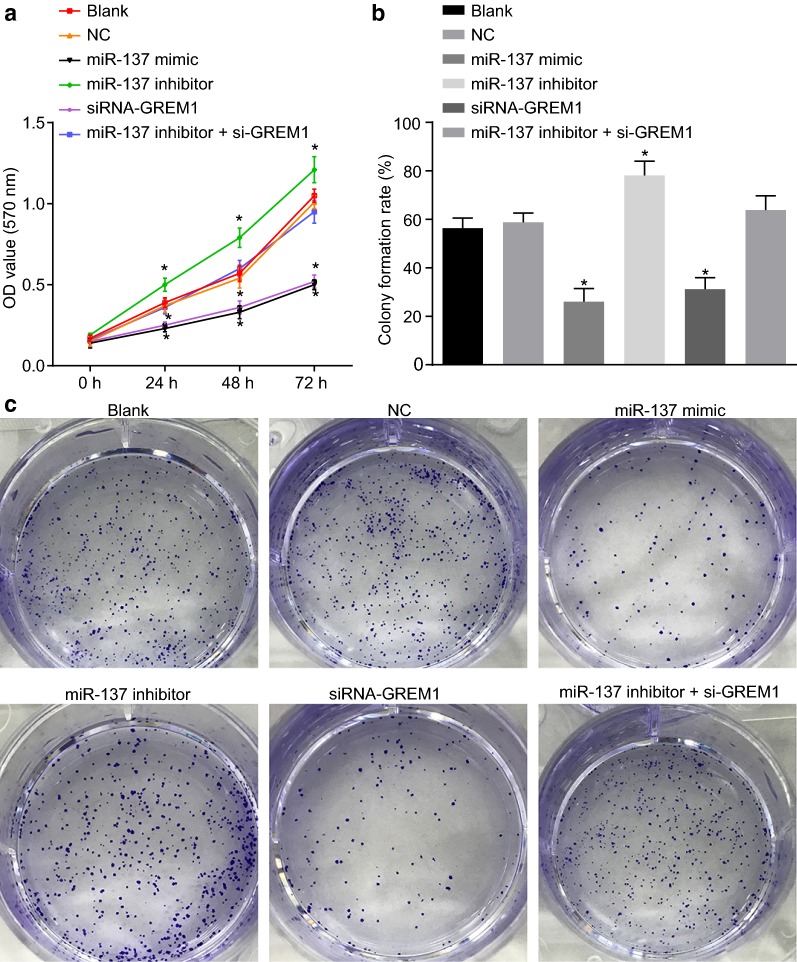
Overexpression of miR-137 reduces CC cell proliferation and colony formation by inhibiting GREM1. **a** The OD value of cells treated with miR-137 mimic, miR-137 inhibitor and/or siRNA-GREM1 at the different time point assessed by MTT assay; **b** colony formation rate of cells treated with miR-137 mimic, miR-137 inhibitor and/or siRNA-GREM1; **c** representative images of colony formation in cells treated with miR-137 mimic, miR-137 inhibitor and/or siRNA-GREM1; *p < 0.05 vs. the blank and NC groups; statistical data were presented as mean ± standard deviation; one-way analysis of variance was used for multi-group comparisons; the experiment was repeated 3 times independently. *CC* cervical cancer, *GREM1* gremlin-1, *miR-137* microRNA-137, *MTT* 3-(4,5-dimethylthiazol-2-Yl)-2,5-diphenyltetrazolium bromide

To further elucidate the effect of miR-137 in CC, we examined its effect on CC cell proliferation by MTT assay (Fig. 7a). Following the treatment of the cells for 24 h, 48 h and 72 h, there was no difference in CC cell proliferation among the blank, NC and miR-137 inhibitor + si-GREM1 groups (*p *> 0.05). The cell proliferation was evidently increased in the miR-137 inhibitor group (*p* < 0.05) and decreased in the miR-137 mimic and siRNA-GREM1 groups (all *p* < 0.05). The colony formation assay (Fig. 7b, c) demonstrated no difference in cell colony formation rate among the blank, NC and miR-137 inhibitor + si-GREM1 groups (*p *> 0.05), while that in the miR-137 inhibitor group was notably higher (*p* < 0.05). In the miR-137 mimic group and the si-GREM1 group, the colony formation rate was lower than that in the blank group and the NC group (*p* < 0.05). The above-mentioned results indicate that miR-137 can suppress CC cell proliferation through the inhibition of GREM1.

### Overexpression of miR-137 enhances CC cell apoptosis and cell cycle arrest by inhibiting GREM1

**Fig. 8:**
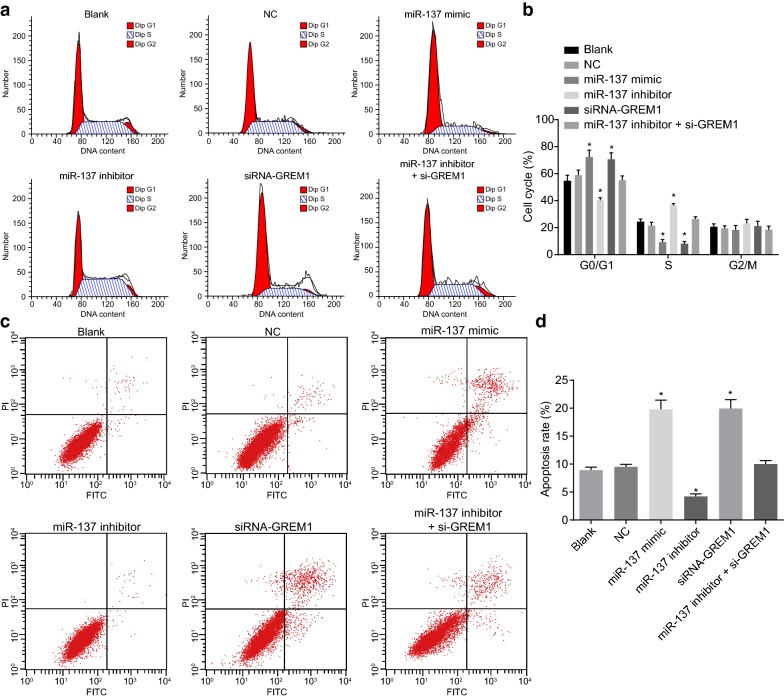
Overexpression of miR-137 enhances the CC cell apoptosis and cell cycle arrest by binding to GREM1. **a** Cell cycle distribution in each group tested by flow cytometry; **b** the statistical analysis of cell cycle distribution; **c** cell apoptosis was measured by flow cytometry; **d** the statistical analysis of cell apoptosis rate; *p < 0.05 vs. the blank and NC groups; statistical data were presented as mean ± standard deviation; one-way analysis of variance was used for multi-group comparisons; the experiment was repeated 3 times independently. *CC* cervical cancer, *GREM1* gremlin-1, *miR-137* microRNA-137

Flow cytometry was conducted to identify whether miR-137 could affect the cell cycle (Fig. 8a, b) and apoptosis (Fig. 8c, d) of CC cells. There was no notable difference in cell cycle distribution among the blank, NC and miR-137 inhibitor + si-GREM1 groups (*p *> 0.05). In the miR-137 mimic and siRNA-GREM1 groups, the greater majority of the cells were arrested at the G0/G1 phase, with increased number of G1 phase arrested cells and decreased number of S phase arrested cells (*p* < 0.05). In the miR-137 inhibitor group, there was a decline in the number of cells arrested in the G1 phase, and an elevation in the number of cells arrested in S phase (all *p* < 0.05), with no significant difference in the percentage of G2/M phase arrested cells among all groups (*p* > 0.05). At the same time, no notable difference in CC cell apoptosis was revealed among the blank, NC and miR-137 inhibitor + si-GREM1 groups (*p *> 0.05). As compared with the blank and NC groups, there was a notable decrease in cell apoptosis in the miR-137 inhibitor group (*p *<0.05), which was increased in the miR-137 mimic and siRNA-GREM1 groups (*p *<0.05). The aforementioned findings illustrated that miR-137 can enhance CC cell apoptosis by inhibiting GREM1.

### Overexpression of miR-137 represses CC cell invasion and migration by binding to GREM1

**Fig. 9:**
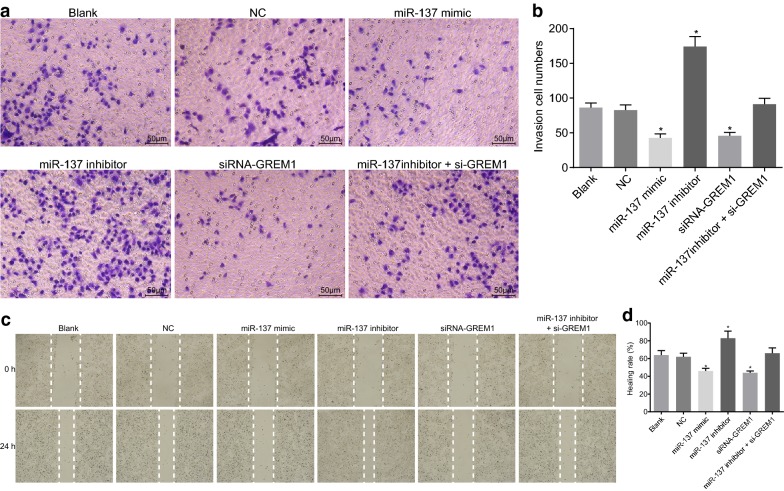
Overexpression of miR-137 inhibits CC cell invasion and migration by binding to GREM1. **a** Cell invasion images using Transwell assay (×200); **b** statistical results of the number of invading cells of Transwell; **c** cell migration distance at 24 h and 48 h (×200); **d** statistical results of the migration distance; *p < 0.05 vs. the blank and NC group; statistical data were presented as mean ± standard deviation; one-way analysis of variance was used for multi-group comparisons; the experiment was repeated 3 times independently. *CC* cervical cancer, *GREM1* gremlin-1, *miR-137* microRNA-137

Transwell assay was conducted to evaluate the effect of miR-137 on CC cell invasion (Fig. 9a, b). No significant difference was detected in CC cell invasion among the blank, NC and miR-137 inhibitor + si-GREM1 groups (*p *> 0.05); however, it was evidently increased in the miR-137 inhibitor group (*p *< 0.05) and decreased in the miR-137 mimic and siRNA-GREM1 groups (*p *< 0.05). It could be concluded that miR-137 inhibited CC cell invasion by inhibiting GREM1.

Next, scratch test was performed to confirm the effect of miR-137 on CC cell migration (Fig. 9c, d). The difference in cell migration wasn’t significantly different among the blank, NC and miR-137 inhibitor + si-GREM1 groups (*p *> 0.05). However, versus the blank and NC groups, the cell migration displayed a notable increase in the miR-137 inhibitor group (*p *<0.05) and a decrease in the miR-137 mimic and siRNA-GREM1 groups (all *p *<0.05). These findings indicated that overexpression of miR-137 could inhibit CC cell migration by inhibiting GREM1.

### Overexpression of miR-137 impedes tumor growth by binding to GREM1 in vivo

**Fig. 10:**
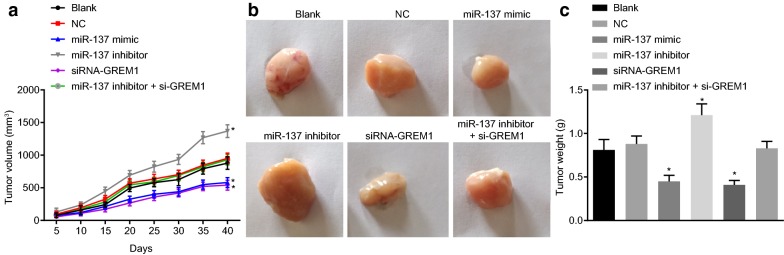
Overexpression of miR-137 inhibits tumor growth by binding to GREM1 in vivo. **a** The tumor volume changes in nude mice; **b** representative tumor images and weight of nude mice in each group; **c** The tumor weight changes in nude mice; *p < 0.05 vs. the blank and NC groups; statistical data were presented as mean ± standard deviation; one-way analysis of variance was used for multi-group comparisons; the experiment was repeated 3 times independently. *GREM1* gremlin-1, *miR-137* microRNA-137, *NC* negative control

The xenograft model in nude mice was developed to evaluate the effect of miR-137 binding to GREM1 gene in vivo. During the first 20 day, there was no significant change in the volume of tumor in nude mice. However, from the 20th day after the injection, the tumor volume began to change, and continuously increased until the 40th day, when the nude mice were treated. As displayed in Fig. 10b, tumor volume didn’t differ notably among the miR-137 inhibitor + si-GREM1, blank and NC groups (*p *> 0.05). The tumor volume in the miR-137 inhibitor group was larger (*p* < 0.05), and that in the miR-137 mimic and siRNA-GREM1 groups was smaller than that in the blank and NC groups (*p* < 0.05). As shown in Fig. 10c, we found no significant difference in tumor weight among the miR-137 inhibitor + si-GREM1, blank and NC groups (*p* > 0.05). In the miR-137 inhibitor group, the tumor weight was heavier (*p* < 0.05), and that was lighter in the miR-137 mimic group and the siRNA-GREM1 group (*p* < 0.05). The above results suggested that miR-137 could hinder tumor growth in nude mice by down-regulating GREM1 expression.

### Overexpression of miR-137 repressed cell invasion and migration via blockage of the TGF-β/smad pathway

**Fig. 11:**
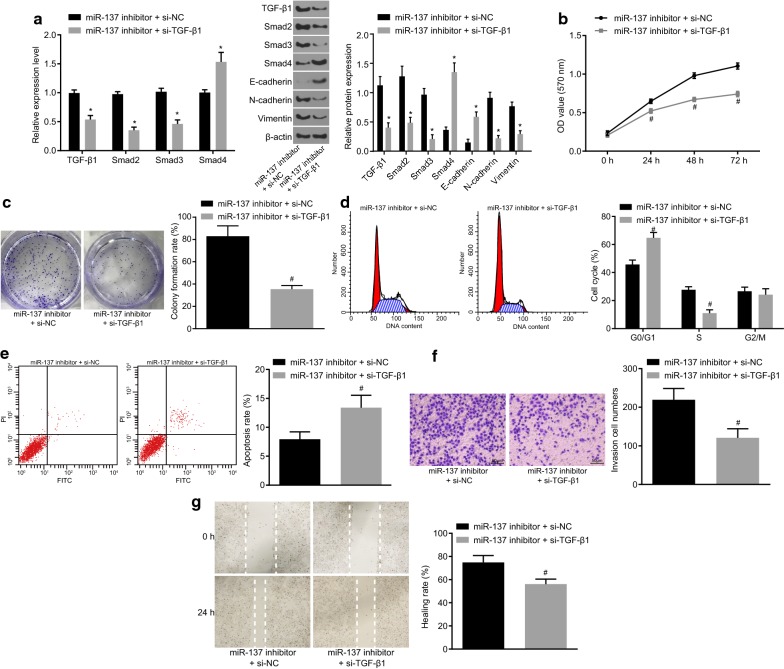
Inhibition of the TGF-β/smad pathway represses CC cell EMT, invasion and migration induced by miR-137 down-regulation. **a** The expression of TGF-β1, Smad2, Smad3, Smad4, E-cadherin, N-cadherin and Vimentin in CC cells co-transfected with miR-137 inhibitor and si-TGF-β1 or si-NC as a control determined by RT-qPCR and western blot analysis; **b** the OD value of CC cells co-transfected with miR-137 inhibitor and si-TGF-β1 or si-NC as a control measured by MTT assay; **c** colony formation situation of CC cells co-transfected with miR-137 inhibitor and si-TGF-β1 or si-NC as a control assess by colony formation assay; **d** the cell cycle distribution of CC cells co-transfected with miR-137 inhibitor and si-TGF-β1 or si-NC as a control tested by flow cytometry; **e** the cell apoptosis of CC cells co-transfected with miR-137 inhibitor and si-TGF-β1 or si-NC as a control tested by flow cytometry; **f** cell invasion of CC cells co-transfected with miR-137 inhibitor and si-TGF-β1 or si-NC as a control using Transwell assay (×200); **g** cell migration distance at 0 h and 24 h using scratch test; ^#^*p* < 0.05 vs. the miR-137 inhibitor + si-NC group; statistical data were presented as mean ± standard deviation; data between two groups were analyzed by paired *t*-test; the experiment was repeated 3 times independently. *CC* cervical cancer, *GREM1* gremlin-1, *miR-137* microRNA-137, *NC* negative control

CC cells were delivered with miR-137 inhibitor + si-NC or miR-137 inhibitor + si-TGF-β1 in order to validate the function of TGF-β/smad pathway in CC cells (Fig. 11). Versus the miR-137 inhibitor + si-NC group, the mRNA and protein level of TGF-β/smad pathway-related factors (TGF-β1, Smad2 and Smad3), and EMT-related proteins (N-cadherin and Vimentin) was found to be reduced, while that of TGF-β/smad pathway-related factors (Smad4) and EMT-related protein E-cadherin increased in the miR-137 inhibitor + si-TGF-β1 group (*p* < 0.05). The cell proliferation, colony formation rate and the ability of cell invasion and migration were suppressed, and the number of cells in G1 phase was elevated, and that of S phase arrested cells was decreased, accompanied by increased rate of apoptosis (*p* < 0.05). These findings provide evidence that blockade of the TGF-β/smad pathway could reverse the promotive effect of miR-137 inhibition on CC progression.

## Discussion

Numerous studies have provided evidence on the ability of various miRNAs in suppressing the growth of CC, such as miR-372, miR-214 and miR-7 [21–23]. Furthermore, the TGF-β/Smad pathway has a critical role to play in the tumor microenvironment, thus mediating cancer progression [24, 25]. The present study provided a new insight suggesting that miR-137 regulated the TGF-β/Smad pathway by binding to GREM1, resulting in the suppression of the migration and invasion of CC cells.

Initially, our results found that in CC tissues and cells, EMT was active, miR-137 was down-regulated, GREM1 was up-regulated and TGF-β/smad pathway was activated. In a recent study, it was found that miR-137 expression was evidently down-regulated in colorectal cancer (CRC) cell, which suggested the involvement of miR-137 in CRC development [26]. It has been established that TGF-β pathway is activated in CC, which was consistent with our results [27]. A high expression in GREM1 has been demonstrated in mesothelioma cells, which induces mesothelioma cell growth [28]. Moreover, GREM1 has been confirmed to be closely related to TGF-β/smad pathway in glioma, colon cancer, chronic pancreatitis, as well as renal damage [29–32]. Besides, TGF-β/smad signaling pathway was inactivated in response to a decrease in GREM1 expression.

In the subsequent experiments, we identified that the effect of miR-137 on EMT was achieved via the TGF-β/smad pathway by binding to GREM1. In response to GREM1 suppression or miR-137 overexpression, a reduction could be detected in EMT of the CC cells. The tumor-suppressive function of miR-137 has been documented in a variety of human tumors, such as colorectal cancer and breast cancer [33, 34]. Furthermore, recent evidence has indicated that miR-137 could diminish the expression of Vimentin and N-cadherin and elevate that of E-cadherin in breast cancer cells, indicating that the overexpression of miR-137 may lead to the suppression of EMT [35]. In line with our findings, a previous investigation demonstrated that the protein expression of N-cadherin and Vimentin are markedly downregulated in GREM1-silenced cells, suggesting that GREM1 may enhance the process of EMT [36]. GREM1 triggers an instant activation of the Smad pathway featured by elevated phosphorylation of Smad3 and Smad2 proteins [37]. Another study revealed that GREM1 is a downstream TGF-β1 mediator that acts to intensify EMT in renal cells as the inhibition of GREM1 decreases TGF-β-promoted EMT [38]. In addition, we also demonstrated that miR-137 could repress the CC cell proliferation, migration and invasion and induce cell cycle arrest and apoptosis via the TGF-β/smad pathway by binding to GREM1. These results were further confirmed by in vivo xenograft tumor formation experiments in nude mice. Consistent with our study, a study has found that Let-7a, could suppress CC cell proliferation by decreasing the expression of TGF-β1 through the TGF-β/smad pathway [39]. Hence, based on the aforementioned findings, miR-137 could potentially serve as a biomarker to inhibit the growth of CC cells.

## Conclusion

**Fig. 12:**
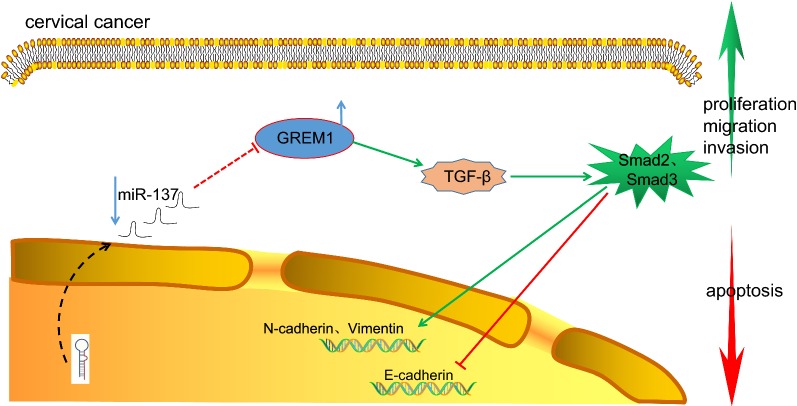
Schematic representation of the underlying mechanism of miR-137 in CC. In CC, the expression of miR-137 decreased significantly and the expression of GREM1 increased significantly. MiR-137 can target and inhibit the expression of GREM1 gene. Inhibition of miR-137 could promote the expression of GREM1, and GREM1 could promote the expression of TGF-β1, Smad2, Smad3, N-cadherin and Vimentin. Overexpression of miR-137 could inhibit the CC cell proliferation, migration and EMT while inducing apoptosis. *CC* cervical cancer, *GREM1* gremlin-1, *miR-137* microRNA-137

Interestingly, the tumor suppressor miR-137 could curtail EMT, cell migration and invasion in CC cells via blockade of the TGF-β/smad pathway by binding to GREM1 (Fig. 12). Thus, we subsequently propose that miR-137 may be a promising therapeutic target for the treatment of CC. However, due to the limitation in experimental conditions and funds, further research in the future is required to properly understand the mechanism, by which GREM1 is involved in the activation of the TGF-β/smad pathway or GREM1 promoter methylation and its increased expression in CC.

## Additional files

**Additional file 1.** Fold change of gene expression of top 50 genes.

**Additional file 2.** Gene expression of top 50 genes.

## Data Availability

The datasets generated and/or analyzed in the current study are available from the corresponding author on reasonable request.

## Abbreviations

CC: cervical cancer
DEGs: differentially expressed genes
DMSO: dimethyl sulfoxide
EMT: epithelial–mesenchymal transition
FBS: fetal bovine serum
FIGO: International Federation of Gynecology and Obstetrics
GEO: Gene Expression Omnibus
GO: gene ontology
miRNAs: microRNA
miR-137: microRNA-137
NC: negative control
OD: optical density
PBS: phosphate buffered saline
PI: propidium iodide
PVDF: polyvinylidene fluoride
TGF-β: transforming growth factor-β
UTR: untranslated region
